# The Analysis of Hand Movement Distinction Based on Relative Frequency Band Energy Method

**DOI:** 10.1155/2014/781769

**Published:** 2014-11-05

**Authors:** Yanyan Zhang, Gang Wang, Chaolin Teng, Zhongjiang Sun, Jue Wang

**Affiliations:** The Key Laboratory of Biomedical Information Engineering, Ministry of Education, Institute of Biomedical Engineering, School of Life Science and Technology, Xi'an Jiaotong University, 28 Xianning West Road, Xi'an 710049, China

## Abstract

For the purpose of successfully developing a prosthetic control system, many attempts have been made to improve the classification accuracy of surface electromyographic (SEMG) signals. Nevertheless, the effective feature extraction is still a paramount challenge for the classification of SEMG signals. The relative frequency band energy (RFBE) method based on wavelet packet decomposition was proposed for the prosthetic pattern recognition of multichannel SEMG signals. Firstly, the wavelet packet energy of SEMG signals in each subspace was calculated by using wavelet packet decomposition and the RFBE of each frequency band was obtained by the wavelet packet energy. Then, the principal component analysis (PCA) and the Davies-Bouldin (DB) index were used to perform the feature selection. Lastly, the support vector machine (SVM) was applied for the classification of SEMG signals. Our results demonstrated that the RFBE approach was suitable for identifying different types of forearm movements. By comparing with other classification methods, the proposed method achieved higher classification accuracy in terms of the classification of SEMG signals.

## 1. Introduction

Millions of people in the world were amputated every year due to diseases, industrial injuries, traffic accidents, and accidental injuries. They hoped to possess a voluntarily controlled prosthetic limb for retrieving the basic human movement capabilities. At the present time, the key point of the prosthetic system is the selection of the information source of movement control. Surface electromyographic (SEMG) signals are one-dimensional and nonstationary time series which can be noninvasively recorded by using electrodes on the skin surface. These signals are the summation of all motor unit action potential (MUAP) within the pick-up area of the electrodes. Hence they are deeply related with the activities and the functional statuses of muscle and can reflect the activities of the neuromuscular system [1]. Meanwhile, due to the convenience and the noninvasive access of the acquisition of SEMG signals, the SEMG signals have become the most attractive source for myoelectric prostheses currently.

In order to control the myoelectric artificial limb, it is very important to extract the effective features to characterize the SEMG signals [2, 3]. So far, many feature extraction methods have been presented for the classification of SEMG signals. Boostani et al. [4–6] extracted some time-domain features of the SEMG signals from upper limb for the control of myoelectric prostheses, such as amplitude, zero crossings, the integral absolute value, variance, and EMG histogram. Farina and Merletti [7] extracted the frequency-domain features by using Fourier transform method for the pattern recognition of multichannel SEMG signals, such as the mean frequency and the median frequency of power spectrum. Hu and Nenov [8] used the coefficients of multichannel AR model as the features of SEMG signals and the classification accuracy was over 95%. In addition, because the time-frequency analysis methods, such as short-time Fourier transform, Wigner distribution, wavelet transform, and wavelet packet transform, combined the time-domain and the frequency-domain characteristics of the signal, these methods can fully describe the signal and have been widely used in the pattern recognition of the SEMG signals [9, 10]. Englehart et al. [11–13] extracted the feature set of SEMG signals based on wavelet transform and wavelet packet transform to improve the classification accuracy. Orosco et al. [14] focused on arithmetic mean, median, trimmed mean estimators, and ensemble average of several bispectrum estimators for the continuous classification scheme of five upper limb movements applied to a myoelectric control of a robotic arm and they have achieved a very good classification performance. Arjunan et al. [15, 16] extracted the correlation dimension and the fractal dimension as the features of SEMG signals for the pattern recognition of the fine-motor performance of human hand.

In recent years, the feature extraction methods of multichannel SEMG signals mostly focused on the combination of the two or more methods mentioned above [5, 6, 12, 13, 17]. Those combined features can enhance the recognition accuracy of SEMG signals theoretically. However, because some movements inaccurately recognized in a control system for powered prostheses would seriously hurt the amputee and result in a frustrated feeling to the user, it is very essential to investigate the extraction method of effective features from the SEMG signals in order to further improve the classification accuracy. In this study, the relative frequency band energy (RFBE) method based on wavelet packet decomposition was proposed to extract features of SEMG signals for the prosthetic pattern recognition. This approach can provide a simple and effective feature extraction of SEMG signals for the myoelectric prosthetic control system.

## 2. Materials and Methods

### 2.1. Subjects and Data Acquisition

A telemetric EMG system (TeleMyo 2400T, Noraxon Inc., Scottsdale, AZ, USA) was used for SEMG data acquisition. The sampling rate of the signals was 1500 Hz for each channel. Differential amplifiers with bandpass filters between 10 and 500 Hz were used to reduce the effects of high frequency noises and low frequency movement artifacts. Four-channel SEMG signals were collected from the extensor digitorum, extensor carpi radialis, palmaris longus, and flexor carpi ulnaris of the right forearm by using four pairs of differential silver-silver chloride disc electrodes (5 mm in diameter) with a center-to-center spacing of 20 mm. The reference electrode was attached to the elbow of a subject. In addition, because the SEMG signals were relatively weak and can be easily submerged in the noise, the exfoliating cream was used to clean the surface skin of forearm of each subject before experiments to reduce the interference generated by the skin impedance.

**Figure 1:**
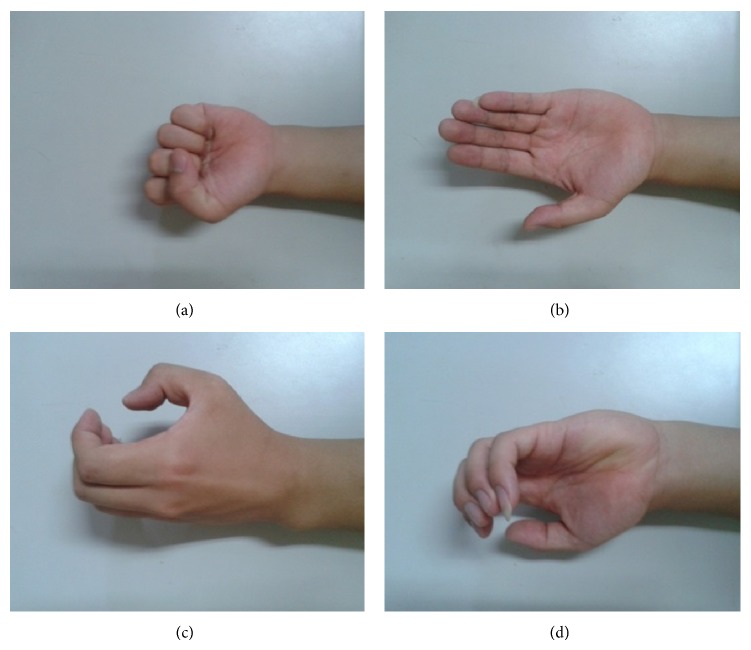
Four different types of movements: (a) hand close, (b) hand open, (c) forearm pronation, and (d) forearm supination.

For the purpose of data acquisition, seven healthy adults voluntarily accepted to participate in this experiment. The mean age of all subjects was 22 (±2) years. As shown in Figure 1, they were all asked to perform four kinds of movements: forearm pronation (FP), forearm supination (FS), hand open (HO), and hand close (HC). These 4 gestures are presented in this study because they denote the commonly used hand motions in everyday life and are very essential for the prosthetic users. If the prosthetic users can skillfully achieve these basic motions in their daily life, their self-confidence and motivation to take this device will be enhanced and their life quality will be further improved. The subject was asked to perform each desired movement with a comfortable and consistent contraction at a moderate force in four trials. In each trial, one forearm movement was repeated consecutively for 60 times and each movement was held for about 2 s. Therefore, there were 240 groups of four channels of SEMG signals for each subject and 1680 groups in total. In addition, in order to avoid the effects of muscle fatigue caused by the long-time contraction during the experiment, the subject was allowed to relax for 3 s between each movement and for 5 min between each of the 20 groups of movements.

The Muscle Research Center at Boston University has discovered that the spectra of SEMG signals were distributed within 20–500 Hz, mostly concentrated in 50–150 Hz, when the bipolar model was used to collect the SEMG signals [18]. In addition, the used frequency band was relatively wide during the data acquisition. Hence, the four-order Butterworth filter was employed to preprocess the four channels of SEMG signals. The SEMG signals were band-pass filtered between 5 and 200 Hz for the pattern recognition of prosthetic movements.

### 2.2. Feature Extraction

For the pattern recognition of prosthetic limbs based on the SEMG signals, it is very critical to effectively extract the features of SEMG signals. In this study, the RFBE method based on wavelet packet decomposition was proposed for the feature extraction of different movements of multichannel SEMG signals. The computation method of the RFBE is then presented as described below.

Assuming that the original SEMG signal is a finite energy signal and is decomposed to the level *J* using wavelet packet transform, the Symlet wavelet of order 5 was selected as the mother wavelet. If the depth of wavelet analysis is too small, the classification information would not be extracted efficiently. If the depth is too large, the computation complexity would be increased remarkably. Therefore, in this study, every SEMG signal was decomposed to the third level using wavelet packet transform. Let *i* denote the channel index of SEMG signals and *W*_*i*,*j*_^*m*^  (*i* = 1,…, 4; *j* = 0,…, *J*; *m* = 0,…, 2^*j*^ − 1) denote the decomposed subspace corresponding to node (*j*, *m*); *j* is the decomposition level and *m* is the index of the subspace occurring at the *j*th level. Hence, the wavelet packet coefficients of the subspace *W*_*i*,*j*_^*m*^ can be described as

(1) $$\begin{array}{c} d_{i,j}^{m}=\{d_{i,j}^{m}\left(k\right)\;|\;k=1,2,\ldots ,K_{j}^{m}\}, \end{array}$$

where *K*_*j*_^*m*^ is the size of the subspace corresponding to node (*j*, *m*) in the wavelet packet decomposition. Because wavelet packet basis functions are orthogonal to each other, the energy in each subspace is defined as

(2) $$\begin{array}{c} E_{i,j,m}=\underset{k}{\sum }{\left|d_{i,j}^{m}\left(k\right)\right|}^{2}. \end{array}$$

Then the total energy of four-channel SEMG signals in the same subspace or frequency band can be calculated by

(3) $$\begin{array}{c} \text{T}{\text{E}}_{j,m}=\underset{i}{\sum }E_{i,j,m}. \end{array}$$

In the end, the RFBE of four-channel SEMG signals in each frequency band is given by

(4) $$\begin{array}{c} P_{i,j,m}=\frac{E_{i,j,m}}{\text{T}{\text{E}}_{j,m}}. \end{array}$$

### 2.3. Feature Selection

In this study, the SEMG signals were decomposed to the third level using wavelet packet transform. For each forearm movement, a 32-dimensional feature vector can be obtained. In order to improve the generalization capability of the classifier and facilitate its design [19, 20], the feature selection should be performed to reduce the dimensionality of the original feature vectors and retain the useful feature components for movement classification. In this study, the principal component analysis (PCA) [21] was employed to perform the feature selection. As is well known, the accumulative variance contribution is usually used as the criterion of determining the feature dimensionality during the utility of the PCA. In addition, the class separability of feature space can have an influence on the classification accuracy for pattern recognition. In order to further take into account the class separability of feature space, the Davies-Bouldin (DB) index [22] was also used as the criterion of determining the feature dimensionality when the feature selection was performed. The DB index is an effective cluster validity measurement which is defined as a function of the ratio of the sum of within-cluster scatter to the between-cluster separation. It has been shown that lower values of the DB index indicate a higher degree of cluster separability. Therefore, in order to reduce the dimensionality of the original feature space and assure the high classification accuracy, the first *k* principal components were used to obtain a *k*-dimensional feature space and regarded as the inputs to a classifier for the classification of the SEMG signals. At the same time, the DB index of this feature space should have the minimum value and the accumulative variance contribution of this feature space should be larger than 90%.

### 2.4. Classifier of Movement Distinction

As for the classifier, the support vector machine (SVM) method was used to evaluate the classification effectiveness of the proposed method. Because the SVM classifier is originally designed to solve the problem of binary classification, it cannot be directly applied in multiclass classification which is usually encountered in practice [23]. At present, there are many algorithms which can extend the binary classifier to the multiclass SVM classifier [24, 25]. In this study, we used the one-against-all strategy to implement the multiclass SVM classifier. This classifier needs to construct *k* SVM models where *k* is the number of classes to achieve the multiclass classification. In addition, in order to estimate the performance of the classifier, the classification results were calculated using five-fold cross-validation method [26]. For each subject, the 240 groups of SEMG signals were randomly divided into mutually exclusive fivefold ones of equal size. The fourfold groups were randomly selected from these fivefold ones as a training set and the last one was regarded as a test set. The feature selection was performed on the training set. Then, the SVM classifier based on the radial basis function (RBF) kernel function was trained with the training set. Lastly, the classification accuracy was obtained using the trained classifier and the test set. This process was repeated until each fold was tested. The cross-validation accuracy was calculated as the average of the five individual accuracy measures.

## 3. Results

### 3.1. Extraction of Action SEMG Signals

**Figure 2:**
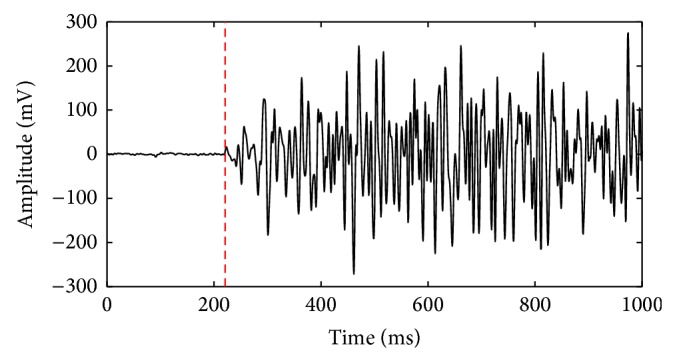
The extraction of one-channel-of-action SEMG signals over the extensor carpi radialis during hand opening. The red vertical line represents the initial timepoint of hand opening.

The extraction of the initial phase of forearm movement is a key issue for the control of prosthetic limbs based on the SEMG signals. In this study, the sliding time window method [27] was applied to detecting of the initial phase of forearm movement. Firstly, the SEMG signals without any actions were used to calculate a threshold value. The length of analysis window is 21 sampling points. The energy values of 25 consecutive windows were averaged to obtain the threshold value. Then, we also used the analysis window with 21 points to sequentially calculate the signal energy. When the energy values of 20 consecutive analysis windows were all greater than the threshold, the middle point of the last analysis window was regarded as the initial timepoint of forearm movement. The extraction of one-channel-of-action SEMG signals over the extensor carpi radialis during hand opening is shown in Figure 2. The red dashed line represents the initial timepoint of forearm pronation which is determined by the sliding time window method. After the initial timepoints of each forearm movement were detected, the SEMG signals with 100, 200, 300, 400, 500, and 600 ms were, respectively, segmented to perform the prosthetic movement pattern recognition.

### 3.2. Determination of the Feature Space

**Figure 3:**
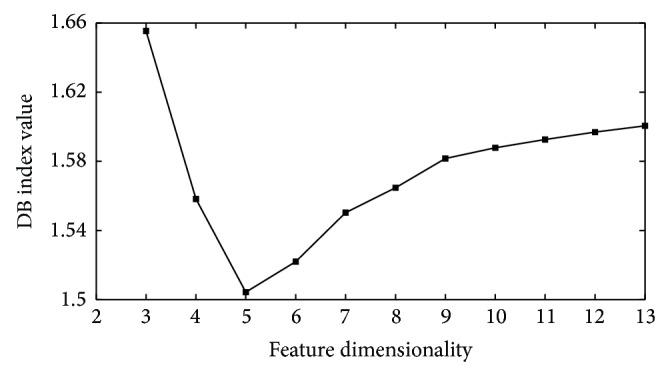
Variation of the DB index with different feature dimensionalities for the SEMG signals of 300 ms in subject 1.

According to the feature selection approach mentioned above, the first *k* principal components which had the minimum value of DB index were chosen to consist of the new feature space of SEMG signals. The DB index values varying with different number of features for subject 1 are illustrated in Figure 3 when the length of SEMG signals is 300 ms. When the feature dimensionality was 5, the DB index reached the minimum value, which represented the maximum degree of the class separability. Therefore, the first 5 principal components were combined to construct the feature vectors for the classification of SEMG signals. In order to further investigate the influence of different data lengths on the classification accuracy of myoelectric prostheses, the SEMG signals with 6 different lengths were used in this study. The feature dimensionalities corresponding to the SEMG signals with different lengths for 7 subjects are summarized in Table 1. As illustrated in this table, the number of used features ranged from 2 to 8, which indicated the validity of the feature selection approach. In addition, there were different numbers of selected features for different subjects in terms of the same data length, which indicated the individual differences of different subjects for the same feature selection algorithm.

### 3.3. Classification Accuracy

Every SEMG signal was decomposed to the third level by wavelet packet transform and the RFBE of SEMG signals in each frequency band was calculated. Because there were four channels of SEMG signals, this resulted in a feature vector including 32 elements. The feature selection was performed by means of the PCA and the DB index criterion. Then, the new feature space was used as the inputs to a nonlinear SVM classifier for the classification of the SEMG signals. The classification accuracy of prosthetic pattern recognition was obtained by the fivefold cross-validation method. The classification results of the different lengths of SEMG signals for 7 subjects are shown in Table 2. The change of classification results for 7 subjects showed the same trends that the classification accuracy was gradually enhanced as the data length of SEMG signals increased. For the SEMG signals of 100 and 200 ms, the standard deviation of classification accuracy was relatively large, which indicated that the difference of classification results was obvious for different subjects when the data length of SEMG signals was short. When the data length was 300 ms, the mean classification accuracy of 7 subjects achieved 93.79% and the standard deviation decreased to 2.74%. In addition, the individual values of classification accuracy of all subjects reached over 90% except subject 3. For the SEMG signals of 600 ms, the highest classification accuracy was 98.81%. When the data length increased from 100 to 300 ms, the classification accuracy was obviously improved. However, while the data length exceeded 300 ms, the classification accuracy of SEMG signals was greater than 90% and the rising trend of classification accuracy became slow.

## 4. Discussion

**Figure 4:**
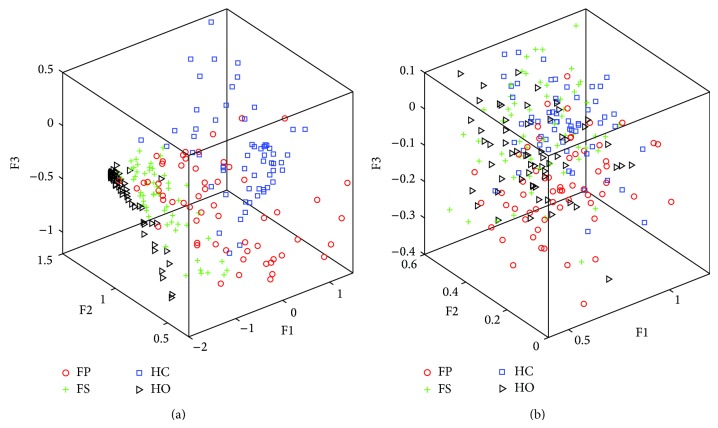
Scatter plot of the RFBE features (a) and the RWPE features (b) of subject 1 for the SEMG signals of 300 ms when the first 3 principal components were used to construct the feature vectors of SEMG signals.

In order to further investigate the performance of the RFBE method, we compared this one with other existing algorithms. The relative wavelet packet energy (RWPE) method was used in this comparison task, which was thought to present the highest classification accuracy for the prosthetic pattern recognition [4]. To compare the performance of both methods, the same training and test sets were applied to the RWPE method. The SEMG signals were decomposed to the third level by wavelet packet decomposition and the RWPE in every subspace was calculated. Then, the feature selection and the classification strategy based on the SVM classifier were performed. The scatter plot of the RFBE and the RWPE of subject 1 for the SEMG signals of 300 ms when using the first 3 principal components to construct the feature vectors of SEMG signals is illustrated in Figure 4. It could be observed that the feature distribution obtained by the RFBE method was relatively concentrated for each class of forearm movements. In addition, the DB index was also used to evaluate the class separability of these two approaches. The values of DB index of the RFBE and the RWPE were 1.504 and 3.902, respectively. The values of classification accuracy of the RFBE and the RWPE were 96.4% and 79.5%. This indicated that the class separability of the feature space obtained by the RFBE method was larger than the RWPE method. Therefore the RFBE method was more suitable for the classification of SEMG signals.

When the RFBE method and the RWPE method are performed for the SEMG signals with different data lengths, the averaged values of DB index across 7 subjects are summarized in Table 3. As shown in this table, the longer the data length was, the smaller the value of DB index was. When the data length was short, the useful classification information of SEMG signals would be lost. This could result in the drop of the class separability. When the data length was long enough, the segmented signals would include sufficient information to classify the SEMG signals. However, if the data length was too long, the computational burden would also be increased remarkably. For each same data length, the DB index of the RFBE method was smaller than that of the RWPE method. This demonstrated that the feature space extracted by the RFBE had greater class separability than that extracted by the RWPE. Hence, the proposed method can improve the classification of SEMG signals.

**Figure 5:**
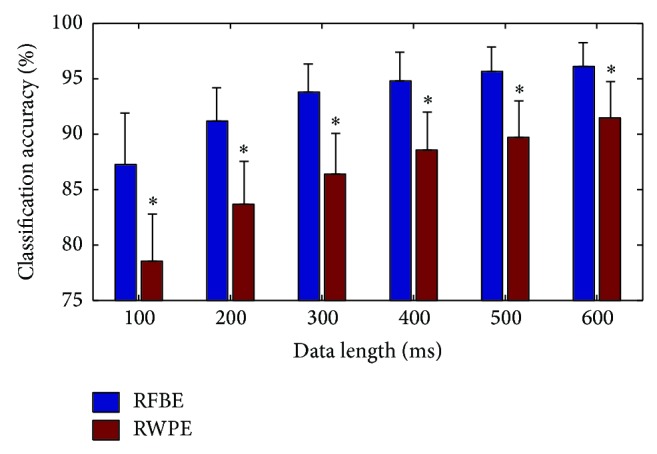
Comparison of mean classification accuracy across seven subjects between the RFBE method and the RWPE method for the SEMG signals with different data lengths. Mean values corresponding to different methods are represented by different color bars. Black bars: standard deviations. An asterisk indicates that the classification results of the RFBE are significantly better than those of the RWPE.

The classification results of the RFBE and the RWPE for the SEMG signals with different data lengths are illustrated in Figure 5. As shown in this figure, the classification results of two methods were both gradually improved as the data length increased. In addition, the classification accuracy using the RFBE method was higher than that of the RWPE method. Then, the one-way analysis of variance (ANOVA) [28] was used to statistically compare the classification results of two approaches. In this study, the significance level was set at *P* < 0.05. The proposed method achieved significant improvement of classification accuracy as compared with the RWPE method for each data length. Thus, the RFBE method was more effective than the RWPE method when the features of SEMG signals were extracted. For the RWPE approach, it can be supposed that the magnitude of signal energy values is distinctly different in various frequency bands. The classification accuracy of the RWPE method is primarily dependent on the frequency band with large signal energy, whereas the RFBE method normalized the energy values of four-channel SEMG signals in every frequency band. Then, the RFBE method made use of all frequency bands to classify the SEMG signals. So the RFBE method can provide more useful information for the control system of powered prostheses than the RWPE method.

When the PCA and the DB index were used to determine the feature space, the DB index was not decreased monotonically while the feature dimensionality increased, as shown in Figure 3. After the feature selection was carried out, the classification information was focused on a few features. When these features that included useful information were used for the classification of SEMG signals, the class separability could achieve the maximum value. If the feature dimensionality further decreased or increased, the class separability would drop because the useful classification information was lost or the noisy information was enhanced.

When using the RWPE method, the highest mean classification accuracy of surface electromyographic (SEMG) signals was achieved by the Symlet wavelet as compared with other wavelets [9]. Hence, we still selected the Symlet wavelet in this study. On one hand, as we know, the wavelet packet transform (WPT) method is a time-frequency analysis tool. The scaling operation in wavelet packet transform can produce a series of basis functions with different window sizes, enabling multiresolution analysis that is suitable for nonstationary signals. In addition, the WPT method can specify the frequency of one signal corresponding to a given time and vice versa. Therefore, the joint time-frequency WPT method provides more useful information for control system for powered prostheses than other methods such as some time domain methods. On the other hand, the waveforms of Symlet wavelet maybe match the SEMG signals and the features extracted by this wavelet can adequately characterize the SEMG signals. So the Symlet wavelet can offer the best classification results for the prosthetic pattern recognition.

In the control system for powered prostheses based on SEMG signals, the classification accuracy and the delay time were regarded as two important criteria on evaluating the classification results of SEMG signals. Therefore, in terms of the SEMG signals with different data lengths, the classification results of the proposed method were compared statistically by the one-way ANOVA. The analysis results are summarized in Table 4. The classification accuracy of 100 ms SEMG signals was significantly lower than those of other data lengths (*P* > 0.05). The classification accuracy of 200 ms SEMG signals was also significantly lower than those of other data lengths (*P* > 0.05) except for the SEMG signals of 300 ms. When the data length ranged from 300 to 600 ms, there was no significant difference of classification accuracy among different data lengths (*P* < 0.05). These demonstrated that the data length of 300 ms can achieve good classification results of SEMG signals and the classification accuracy was not significantly improved after the data length exceeded 300 ms. It is generally considered that a 200 to 300 ms interval is the maximum delay time which can be acceptable for prosthetic users in clinical applications [29], because the long-time delay can make the users frustrated with the slow response of the prosthetic limbs and give up the prostheses. Therefore, to make a compromise between the classification accuracy and the delay time, the data length of 300 ms serves as a suitable choice.

## 5. Conclusions

In this study, the RFBE method based on wavelet packet decomposition was presented to extract the features of SEMG signal for the prosthetic pattern recognition. Compared with other existing methods, the proposed method provided significantly better classification results for each data length of SEMG signals, which demonstrated that the RFBE method was suitable for identifying different types of forearm movements. Additionally, when the data length was 300 ms, the RFBE method obtained good classification results. This satisfied the requirements of the high classification accuracy and the low delay time for the prosthetic control system based on the SEMG signals. The approach proposed in this study will also provide a new idea for analyzing other physiological signals and be easily extended to other applications. The future work will consider recruiting more subjects including the hand amputees into our database. At the same time, more motion classes and more repetitions for each movement class will be also extended for integrating a multifunctional system of prosthetic limbs.

## Figures and Tables

**Table 1:** The feature dimensionalities related to the SEMG signals with different lengths for 7 subjects.

Subject	Data length of SEMG signals (ms)					
	100	200	300	400	500	600
1	8	4	5	4	4	4
2	6	4	5	5	5	5
3	4	2	3	3	3	3
4	7	3	6	5	3	4
5	4	4	4	4	4	4
6	6	6	5	5	6	5
7	8	5	5	5	5	5

**Table 2:** Classification accuracy (%) of the different lengths of SEMG signals for 7 subjects.

Subject	Data length of SEMG signals (ms)					
	100	200	300	400	500	600
1	85.73	92.76	96.40	97.78	98.74	98.52
2	90.39	92.87	93.86	94.71	95.89	95.18
3	91.69	91.67	89.36	90.11	91.84	92.11
4	77.71	85.55	91.80	95.46	95.99	96.73
5	91.61	95.48	97.40	98.23	97.82	98.81
6	84.71	88.40	92.98	92.74	93.37	94.75
7	88.92	91.48	94.72	94.54	95.90	96.54
Mean ± std	87.25 ± 5.02	91.17 ± 3.26	93.79 ± 2.74	94.80 ± 2.81	95.65 ± 2.39	96.09 ± 2.32

**Table 3:** The averaged values of DB index across 7 subjects for the SEMG signals with different data lengths when using the RFBE method and the RWPE method.

Data length (ms)	DB index	
	RFBE	RWPE
100	2.704	4.545
200	1.94	2.980
300	1.685	2.615
400	1.584	2.413
500	1.382	2.165
600	1.371	1.976

**Table 4:** Comparison of the classification results for SEMG signals with different data lengths by using the one-way ANOVA.

Data length 1 (ms)	Data length 2 (ms)	*P* value
100	200	0.029
	300	0.001
	400	0.000
	500	0.000
	600	0.000

200	300	0.138
	400	0.042
	500	0.013
	600	0.007

300	400	0.562
	500	0.287
	600	0.19

400	500	0.623
	600	0.457

500	600	0.799
